# Utilization of social work in adult emergency departments: a scoping review

**DOI:** 10.1007/s43678-025-01082-2

**Published:** 2026-02-19

**Authors:** Christie McLaren, Madeleine Goodwin, Eleni Kirkikis, Linda Rost, Sylvia Salada, Caroline Monnin, Carmen Hrymak, Murdoch Leeies, Anna Reed

**Affiliations:** 1 Department of Emergency Medicine, Max Rady College of Medicine, University of Manitoba, Winnipeg, MB Canada; 2 Department of Social Work, Health Sciences Centre, Winnipeg, MB Canada; 3 Neil John Maclean Health Sciences Library, University of Manitoba, Winnipeg, MB Canada

**Keywords:** Emergency medicine, Social work, Trauma centre, Social emergency medicine, Médecine d’urgence, Travail social, Centre de traumatologie, Médecine d’urgence sociale

## Abstract

**Objectives:**

Emergency departments (EDs) increasingly serve patients with complex social needs alongside medical concerns. Social workers are vital interprofessional team members whom address these needs, though their role varies significantly across institutions. This scoping review explores the role of social work in adult emergency departments as described in current literature.

**Methods:**

Following Joanna Briggs Institute guidelines and PRISMA-ScR reporting standards, we conducted a comprehensive literature search across MEDLINE, PsycINFO, Scopus, and CINAHL, focusing on English-language articles published since 2004 that describe social workers’ direct patient care roles in hospital adult EDs.

**Results:**

Twenty-seven articles met the inclusion criteria, representing diverse geographical contexts and methodologies. Social work roles were clustered into six themes: connecting to community resources (*n* = 13), reducing hospital use and admission (*n* = 6), psychosocial assessment and support (*n* = 6), guiding patients through serious illness (*n* = 5), disposition and safe discharge planning (*n* = 4), care planning and case management (*n* = 4). When reported, staffing models included weekday coverage (*n* = 2), extended hours (*n* = 5), and 24/7 coverage (*n* = 5).

**Conclusion:**

Social workers fulfill diverse roles in EDs, primarily focused on referrals to community resources, reducing hospital utilization, providing psychosocial support, and guiding patients through serious illness. Significant variation exists in implementation, staffing, and utilization of social work across institutions. EDs should consider involving social work directly with critically ill patients, as well as supporting the improvement and development of relationships with community services.

**Supplementary Information:**

The online version contains supplementary material available at 10.1007/s43678-025-01082-2.

## Résumé

**Objectifs:**

Les services d’urgence (SU) servent de plus en plus les patients ayant des besoins sociaux complexes aux côtés des préoccupations médicales. Les travailleurs sociaux sont des membres d’équipe interprofessionnels essentiels pour répondre à ces besoins, mais leur rôle varie considérablement selon les institutions. Cette revue explorera le rôle du travail social dans les services d’urgence pour adultes tel que décrit dans la littérature actuelle.

**Méthodes:**

En suivant les directives de l’Institut Joanna Briggs et les normes de rapport PRISMA-ScR, nous avons effectué une recherche documentaire complète à travers MEDLINE, PsycINFO, Scopus et CINAHL, se concentrant sur des articles en langue anglaise publiés depuis 2004 qui décrivent les rôles des travailleurs sociaux dans les soins directs aux patients dans les SU adultes à l’hôpital.

**Résultats:**

Vingt-sept articles ont répondu aux critères d’inclusion, représentant des contextes géographiques et méthodologies divers. Les rôles en travail social ont été regroupés en six thèmes : se connecter aux ressources communautaires (*n*=13), réduire l’utilisation et l’admission à l’hôpital (*n*=6), évaluation psychosociale et soutien (n=6), guider les patients à travers des maladies graves (*n*=5), disposition et planification de sortie sécuritaire (*n*=4), planification des soins et gestion de cas (*n*=4). Lorsqu’ils ont été déclarés, les modèles de dotation comprenaient la couverture des jours de semaine (*n*=2), les heures prolongées (*n*=5) et la couverture 24/7 (*n*=5).

**Conclusion:**

Les travailleurs sociaux remplissent divers rôles dans les services d’urgence, principalement axés sur l’aiguillage vers des ressources communautaires, la réduction de l’utilisation des hôpitaux, le soutien psychosocial et l’orientation des patients atteints de maladies graves. Il existe des variations importantes dans la mise en œuvre, la dotation et l’utilisation entre les institutions. Les résultats pour le patient et le département pourraient être améliorés en employant efficacement les rôles de travail social et en mettant l’accent sur des interventions de travail social basées sur les compétences.

## Clinician’s capsule

**Table:** 

***What is known about the topic?***
Emergency Departments increasingly serve patients with complex social needs alongside medical concerns.
***What did this study ask?***
This scoping review explored the role of social work in adult Emergency Departments as described in the current literature.
***What did this study find?***
Six main Emergency Department social work roles were identified, including connecting to community resources, reducing hospital use, psychosocial support, guiding through serious illness, discharge planning, and case management.
***Why does this study matter to clinicians?***
Our findings detail how utilizing social work effectively can lead to improved clinical outcomes for patients and department use.

## Introduction

Emergency departments (EDs) are the first point of care for many patients, treating diverse illnesses and conditions [1]. However, globally, EDs are in a state of crisis [2]. In addition to long wait times and overcrowding [2], EDs increasingly manage patients with complex medical and psychosocial needs, including growing numbers of low-income and marginalized patients seeking care for non-urgent concerns [2–4]. To address these psychosocial needs, implementing social workers in EDs has become critical in supporting physicians and nurses with emergent patient care [2, 5, 6].

While social work has expanded to become standard in many EDs [4], the role remains variable across jurisdictions [1, 4]. While existing research has identified a comprehensive range of social work consult services, including psychosocial assessment, advocacy, cultural liaison, addiction support, reporting abuse, and resource mobilization [1, 7]. According to Selby et al. [1] further research is important as existing studies suggest that ED social workers can be under-utilized, and some physicians are unsure how to involve social workers in ED care. However, to our knowledge, no recent comprehensive literature review of ED social work roles has been conducted. In doing so, this scoping review can better equip clinical teams in navigating the growing psychosocial needs facing our EDs and help to identify any gaps in their current standards of ED social work practice.

This review aimed to explore the role of social work in adult EDs, as described in the current literature. Secondarily, this review intended to identify different social work staffing models currently being used in EDs.

## Methods

A scoping review methodology was selected to map key concepts and identify knowledge gaps. This review followed Joanna Briggs Institute guidelines [8] and was reported in accordance with the Preferred Reporting Items for Systematic Reviews and Meta‐Analysis Extension for Scoping Reviews (PRISMA‐ScR) [9, 10]. The protocol was registered in Open Science Framework in April 2024: https://osf.io/48dey.

### Search strategy

A peer reviewed [11] search strategy was conducted by a health sciences librarian (CM) in August 2023 in MEDLINE (Ovid; 1946-August 24, 2023), Psycinfo (Ovid; 1806-August Week 3, 2023), Scopus (Elsevier; -2023), and CINAHL with Full Text (Ebscohost; 1981–2023) (for full search details see Appendix A (Table 1, Table 2, Table 3, Table 4 and Table 5)). Records were limited to English only. The search used controlled vocabulary (i.e. MeSH) and keywords related to the terms related to ‘Social Work’ and ‘Emergency Department'. Grey literature was searched through Advanced Google for hospital guidelines or policies, in addition to a search for theses in ProQuest Dissertations & Theses. All records were exported into Endnote (version × 20; Clarivate Analytics, Philadelphia, PA, USA) and duplicates were removed [12]. Search results were exported to Covidence (Melbourne, Australia).

**Table 1:** Special populations targeted by social work interventions

Population targeted by social work intervention	Number of studies
Non-specific ED population	7
Gender based violence	5
Frequent ED users	4
Addiction	3
Human immunodeficiency viruses	2
Palliative and serious illness	2
Mental health	2
Traumatic brain injury	1
Older adults	1

**Table 2:** Social work roles emphasized

Role of Social Work Emphasized	Number of studies
Connecting to community resources	13
Reducing hospital use and admission	6
Psychosocial assessment and support	6
Guiding patients through serious illness	5
Disposition and safe discharge planning	4
Care planning and case management	4
Did not speak to specific social work role, discussed general scope of social work practice in the ED	4

### Inclusion criteria

Articles were included if they discussed the role of social workers practicing directly in adult ED settings and detailed direct patient care interventions or consult types. Articles were excluded if they focused on social work education, attitudes, or proposed practices only, or if they related to pediatric, outpatient, or community care exclusively. Articles published prior to 2004 were excluded.

### Article selection and data extraction

Article screening was conducted through Covidence software. Each article was reviewed twice by two of six independent reviewers (AR, MG, EK, LR, and CM), in both the title and abstract screening stage, as well as the full-text review stage. For any screening conflicts that arose, a final consensus was reached by a third reviewer not involved in the original conflict.

Data from the included articles were extracted using a standardized form into the following areas: author(s), year of publication, country, type of study and methodology, characteristics of social work role, and staffing. Data extraction was split between six independent reviewers (AR, MG, EK, LR, and CM). A critical appraisal was not completed, as per current scoping review methodology guidance [8, 9].

## Results

**Fig. 1:**
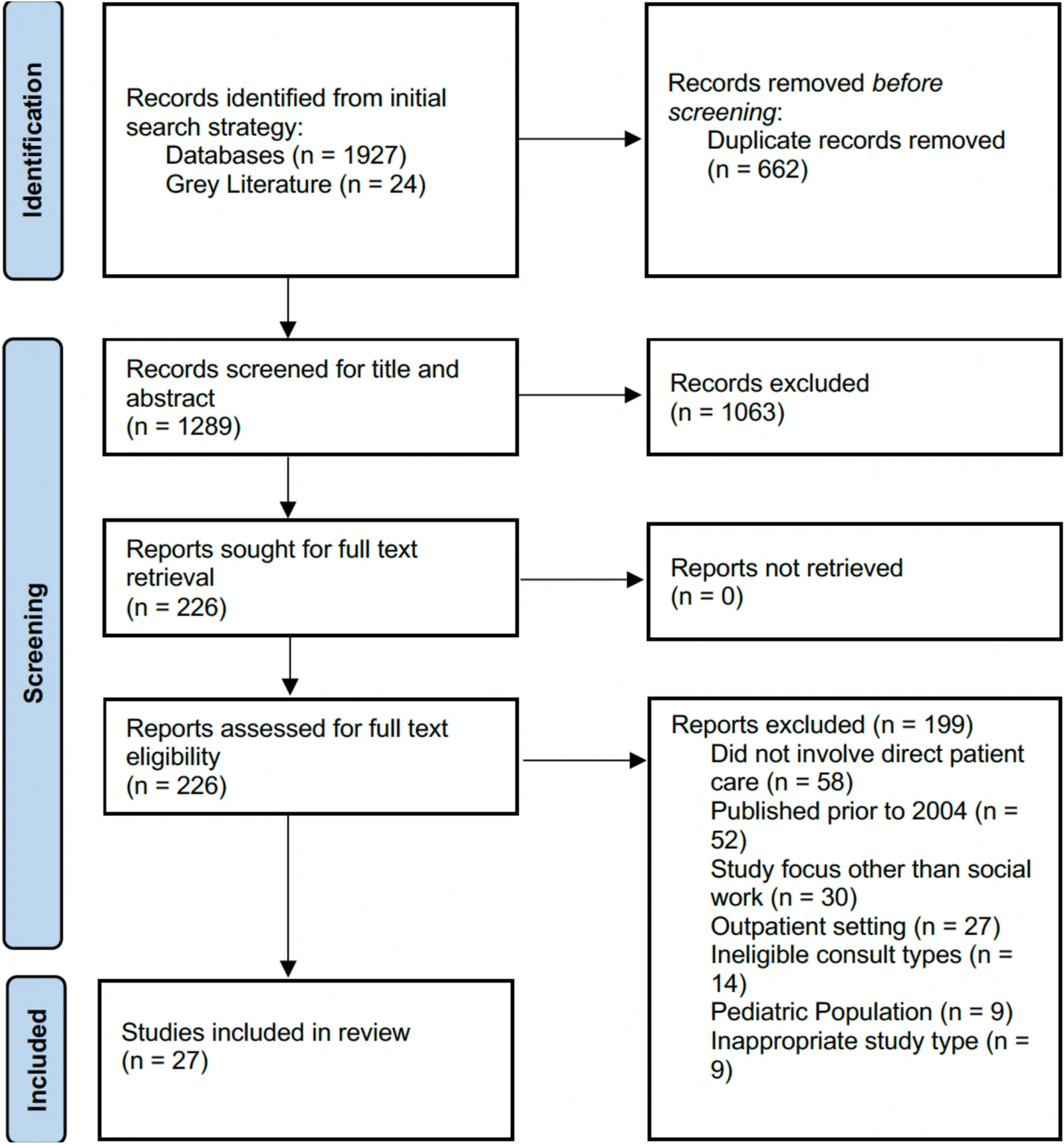
Flow diagram

In total, 1951 articles were identified with the initial search strategy, 662 duplicates were removed, leaving 1289 for screening. After title and abstract screening, as well as full text review, 27 articles met our search criteria and were included in data extraction (Fig. 1). Countries represented were: United States (*n* = 19), Canada (*n* = 2), Australia (*n* = 3), New Zealand (*n* = 1), Korea (*n* = 1), and Sweden/United Kingdom (*n* = 1). Study types included quantitative studies (*n* = 12), pilot studies (*n* = 8), qualitative studies (*n* = 5), and randomized control trials (*n* = 2).

Many of the studies detailed social work interventions aimed at specific populations, including those experiencing gender-based violence (including intimate partner violence, sexual assault, and human trafficking) (*n* = 5), frequent ED users (*n* = 4), addiction (*n* = 3), human immunodeficiency viruses (*n* = 2), palliative and serious illness (*n* = 2), mental health (*n* = 2), older adults (*n* = 1), and traumatic brain injuries (*n* = 1). Seven studies did not describe a specific population (Table 6).

Only 12 of the 27 studies reported staffing models: weekdays (*n* = 2), extended hours (*n* = 5), and 24/7 coverage in the ED (*n* = 5).

**Fig. 2:**
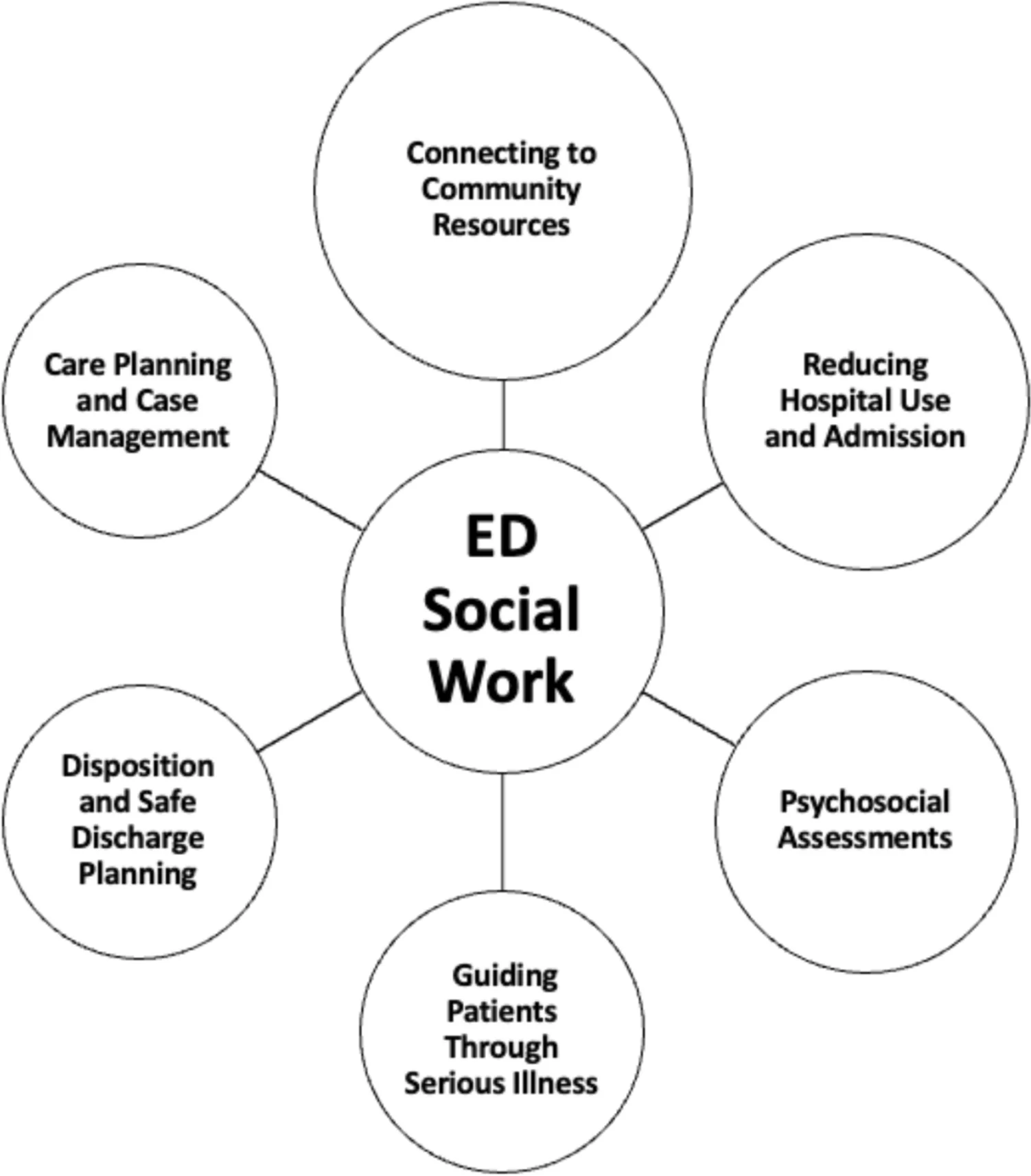
Roles of ED social work

The social work roles identified in the data extraction phase were grouped into six overarching themes (Fig. 2). Understandably though, many of the social work roles described in the various studies often incorporated multiple tasks. Meaning, for example, the ED social worker was sometimes tasked with performing an intervention of some kind, then subsequently was required to organize a discharge plan. As a result, some of the articles fall into more than one overarching theme. In addition, a small number of articles (*n* = 4) did not describe specific social work tasks and instead discussed the role of social workers generally in the ED (see Appendix B for Data Extraction Table) (See Table 7).

### Connecting to community resources (*n* = 13)

Connecting patients to community resources was the most frequently discussed social work role among the final articles. In some cases, social work connected patients to primary care services preventing future ED use. For example, four articles noted that as part of the social work assessment in the ED, the social worker made efforts to link patients to more helpful primary care services in the community [2, 13–15]. Other studies focused on connecting patients to appropriate follow-up support in community, including human immunodeficiency viruses primary care, outpatient treatment for substance use, resources for maintenance of traumatic brain injury, mental health and gender-based violence supports, as well as hospice services [16–24].

### Reducing hospital use and admission (*n* = 6)

Six of the included articles describe part of the social work role in the ED as an attempt to mitigate non-urgent use of ED care, and reduce hospital admissions, when appropriate. For example, in some cases the ED social worker completed their assessment and would make recommendations to the ED physician or other clinical ED team members about whether admission to the hospital was indicated [2, 14, 25, 26]. In other cases, ED social work helped prevent future non-urgent ED visits by connecting the patient with more appropriate resources in the community that could better address their non-urgent and ongoing care needs [13, 27].

### Psychosocial assessments (*n* = 6)

In many studies, the ED social worker’s role was noted to incorporate a tailored psychosocial assessment as part of their role. Four of these studies related to various forms of gender-based violence, including domestic violence and human trafficking. If a patient disclosed abuse during triage or medical assessment, the ED social worker was consulted to review the patient’s case and complete a risk assessment [21, 28–30]. In the remaining two studies, the ED social worker’s assessment focused on the patient’s mental health [19, 25]. One study extended the assessment post-discharge, with social workers providing four follow-up sessions focusing on their wellbeing, medication management, and outpatient medical follow-up [19]. In another study, the ED social worker’s psychosocial assessment, inclusive of family interviews, contributed to the decision with respect to admission to psychiatric unit [25].

### Guiding patients through serious illness (*n* = 5)

Social workers facilitated serious illness conversations using structured guides to discuss goals of care and clarify cardiopulmonary resuscitation status [31, 32]. In addition, the ED social worker provided support to patients presenting to the ED with complex illness, including human immunodeficiency viruses and traumatic brain injury [16, 17, 20]. Two studies described the ED social worker as supporting patients at risk for, as well as being diagnosed with, human immunodeficiency viruses [16, 17]. The ED social worker would support the ED physician with result notification and then remain with the patient to discuss what to expect with their new diagnosis, detailing the public health process, partner notification, and ongoing medical care [16, 17]. In another intervention, the ED social worker provided education to patients with new traumatic brain injuries as part of the “Social Work Intervention for Mild Traumatic Brain Injury (SWIFT-Acute)” [20]. The social worker would discuss brain injury symptoms, coping strategies, substance use, recovery process, as well as informing the patient of community and medical resources for follow-up [20].

### Disposition and safe discharge planning (*n* = 4)

In some of the studies, the ED social workers role incorporated discharge planning directly from the ED [22, 25, 26, 33]. For example, for patients who presented to the ED after experiencing a form of gender based violence, the ED social worker was responsible for obtaining appropriate safe shelter space for the patient [33]. Should the ED social worker have been unable to obtain a safe shelter space, the patient was placed in an observation unit for further safe discharge support [33]. ED social workers also arranged appropriate hospice services at time of discharge from the ED [22]. In collaboration with a case manager, the ED social worker would arrange transportation, discharge location, required equipment, and any other necessary support [22]. Finally, ED social workers, after assisting with a psychiatric assessment, were responsible for sharing assessment findings with interdisciplinary ED team to determine an appropriate discharge plan for patient, should the patient not be admitted to the hospital [25].

### Care planning and case management (*n* = 4)

ED social workers were responsible for managing and developing ED-driven care plans, as well as performing case management. Two of these studies described ED care plans for patients frequently presenting to the ED [15, 27]. The ED social worker was responsible for developing and implementing care plans, as well as ongoing patient support [15, 27]. The two remaining studies described short-term case management [19, 34]. In one study, the ED social worker was part of the ED High User Case Management team, which offered unhoused patients (with four or more ED visits within a 6-month period) short-term intensive case management [34]. Whereas, the remaining study described ED social workers providing short-term support following discharge for patients who had presented to the ED following a suicide attempt [19]. The ED social workers would complete four sessions with patients after discharge before connecting them to mental health resources for further support [19].

### General role of ED social worker (*n* = 4)

Finally, four articles did not speak to a specific social work role or intervention in the ED [1, 7, 35, 36]. Rather, the articles described the ED’s social work role in general, which encompassed the themes identified above. Social workers in the ED were described as advocates, discharge planners, and care coordinators. These social workers addressed concerns related to substance use and addiction, finances, mental health crises, and abuse. They also provided education to ED staff regarding these challenges.

## Discussion

### Interpretation of findings

Our scoping review highlighted six overarching themes which described the roles of social workers practicing in EDs: connecting to community resources, reducing hospital use and admission, psychosocial assessments, guiding patients through serious illness, disposition and safe discharge planning, as well as care planning and case management. Our analysis revealed that the ED social work role can be multifaceted, varied across hospitals, and serves diverse patient populations. In some settings, social workers were embedded within specialized teams (e.g., trauma, geriatric, or bereavement teams) and consulted concurrent with patient presentation. In other cases, social work consultations were initiated following initial nursing or physician assessments, particularly for patients with specific conditions such as human immunodeficiency viruses, or trauma. Staffing models were only reported in 12 articles, with 24/7 and extended coverage most commonly mentioned.

### Comparison to previous studies

To our knowledge, a scoping review in this area has not yet been published. One previous literature review by Moore and colleagues in 2012 found that ED social work research was quite limited [4]. From their available data, the authors were able to describe a few main consult types and the ED social worker’s role in discharge planning. In comparison, we were able to search a much larger body of research in this area, leading to a more robust analysis. Interestingly, at that time, Moore’s review discussed emerging research investigating whether the introduction of social work to EDs would reduce costs or lead to decreased admissions. Our scoping review included many of these studies, which showed that ED social work was beneficial in reducing hospital costs and admissions [2, 25, 26]. Our group suggests that while these initial studies may have been helpful to encourage and justify ED social work to administrators, we argue further research into this area is no longer needed, as we should be aiming to refine and improve the role of social work in the ED, rather than demonstrating its necessity.

### Strengths and limitations

Our study creates new insights into ED social work practice. Using a scoping review methodology, we were able to map out roles, staffing, and utilization trends which had previously not been described. Using this information, we were able to make tangible recommendations for improving patient care through social work involvement.

Our study was limited in that we could not search every available database. As our grey literature search was restricted to hospital guidelines and theses, the omission of conference proceedings is a notable limitation that may have reduced the breadth of sources.

### Health systems implications

Guiding patients through serious illnesses emerged as a major social work role in our review. Our team initially found this result surprising, as it is not emphasized in our local context. However, our review  of these interventions showed that social work involvement in serious illness management has the potential to be highly effective for patients. For example, Edmonds et al. [16] found that social work involvement in every stage of human immunodeficiency viruses care from diagnosis to ongoing treatment found that their program exceeded national averages for patient follow-up, retention, adherence to medications, and viral suppression. Similar success was seen in patients with traumatic brain injury, who, with social work intervention, were more likely to maintain their pre-injury levels of community functioning and significantly reduce their alcohol consumption, compared to those who did not receive social work intervention [20]. Further, we recommend EDs develop and maintain relationships with community agencies that support patients following discharge from the ED. We provide this call to action alongside a recognition that a main constraint noted by ED social workers across included studies was a lack of availability of community resources, as well as barriers to community service referral. ED-based solutions supported by evidence in our review included increasing social work hours in the ED, or social work-specific beds for patients with more complex disposition needs [18, 19, 30, 33]. Finally, a government or health-systems directed policy outlining the role of ED social work may help to guide ED social work teams and align them more closely with outpatient social services. Preliminary success was demonstrated in the UK and Sweden, where healthcare policy assigns ED social workers a key role in diverting older adults from frequent ED visits or admission on social grounds. Older adults in those settings reported ED social work intervention as vital to their short- and long-term health post-ED care [14].

### Research implications

Current research in this area is primarily retrospective cohort studies and/or pilot studies detailing specific interventions or the utility of social work in the ED itself. Given the wide range of social work roles and responsibilities found in our review, our group suggests that further research into the most effective use of social work would be beneficial. Investigating outcomes of different staffing models, duties, and the scope of practice would guide social work ED programs and help to tailor social work education. Additionally, an investigation into the standardization of social work practice in EDs while maintaining flexibility for local contexts would be beneficial. Lastly, patient-centered and/or department-based outcomes are necessary to determine the impact of social work, rather than relying on perceived effectiveness by participants.

## Conclusion

This scoping review demonstrates that social workers fulfill diverse and vital roles in EDs, focused on community resource referral, reducing hospital utilization, completing psychosocial assessment, guiding patients through serious illness, care planning and case management, as well as safe discharge planning. While social work services are clearly valuable in emergency settings, there is significant heterogeneity in how these services are implemented and staffed across institutions. Our findings suggest that involving ED social workers directly with critically ill patients, and improving their ability to connect to community resources and services will lead to improved outcomes for patients and hospitals.

## Supplementary Information

Below is the link to the electronic supplementary material.

Supplementary file1 (DOCX 36 kb)
