# Supplementary material for: Utilization of social work in adult emergency departments: a scoping review
Source: CJEM. 2026 Feb 19;28(8):710–8. doi: 10.1007/s43678-025-01082-2 (PMC13490241; doi:10.1007/s43678-025-01082-2)
Supplement: Supplementary file 1 [file 43678_2025_1082_MOESM1_ESM.docx]

Appendix A. Search strategy as performed in MEDLINE (Ovid), Psycinfo (Ovid), Scopus, and Cinhal with full test (Ebscohost)

Table 1. Medline

| Database: Medline  Platform: Ovid  Date Searched: August 25, 2023  Number of Results: 817 | |
| --- | --- |
| # | Searches |
| 1 | social workers/ |
| 2 | (social worker* or social services worker* or social service worker* or social work team* or social care worker*).tw,kf. |
| 3 | social work/ or social work.tw,kf. |
| 4 | Social Work Department, Hospital/ |
| 5 | Social Work, Psychiatric/og |
| 6 | or/1-5 |
| 7 | exp Emergency Service, Hospital/ |
| 8 | exp Emergency Medicine/ |
| 9 | Emergency Services, Psychiatric/ |
| 10 | ((emergency or trauma) adj2 (department* or unit* or ward* or hospital* or room* or center* or centre*)).tw,kf. |
| 11 | or/7-10 |
| 12 | 6 and 11 |
| 13 | limit 12 to english language |

Table 2. PsycInfo

| Database: PsycInfo  Platform: Ovid  Date Searched: August 25, 2023  Number of Results: 206 | |
| --- | --- |
| # | Searches |
| 1 | social workers/ |
| 2 | (social worker* or social services worker* or social service worker* or social work team* or social care worker*).tw,hw. |
| 3 | social work/ or social work.tw,hw. |
| 4 | exp Emergency Medicine/ |
| 5 | ((emergency or trauma) adj2 (department* or unit* or ward* or hospital* or room* or center* or centre*)).tw,hw. |
| 6 | or/1-3 |
| 7 | or/4-5 |
| 8 | 6 and 7 |
| 9 | limit 8 to english language |
| 10 | limit 9 to ("0200 book" or "0240 authored book" or "0280 edited book" or "0300 encyclopedia" or "0400 dissertation abstract" or "0500 electronic collection") |
| 11 | 9 not 10 |

Table 3. Scopus

Database: Scopus

Date Searched: August 25, 2023

Number of Results: 246

TITLE-ABS-KEY ( social AND worker* OR social AND work OR social AND service AND worker* OR social AND work AND team* OR social AND care AND worker* ) AND TITLE-ABS-KEY ( ( emergency OR trauma ) W/2 ( department* OR unit* OR ward* OR hospital* OR room* OR center* OR centre* ) ) AND ( LIMIT-TO ( LANGUAGE , "English" ) )

Table 4. Cinahl with Full Text

| Database: Cinahl with Full Text  Platform: Ebscohost  Date Searched: August 25, 2023  Number of Results: 658 | |
| --- | --- |
| # | Searches |
| S1 | (MH "Social Workers") OR (MH "Social Worker Attitudes") |
| S2 | (MH "Social Work") OR (MH "Social Work Service") OR (MH "Social Work, Psychiatric") OR (MH "Social Work Practice") |
| S3 | TI ( (social worker* or social services worker* or social service worker* or social work team* or social care worker* or social work) ) OR AB ( (social worker* or social services worker* or social service worker* or social work team* or social care worker* or social work) ) |
| S4 | S1 OR S2 OR S3 |
| S5 | (MH "Emergency Service+") |
| S6 | (MH "Emergency Medicine") |
| S7 | TI ( ((emergency or trauma) W2 (department* or unit* or ward* or hospital* or room* or center* or centre*)) ) OR AB ( ((emergency or trauma) W2 (department* or unit* or ward* or hospital* or room* or center* or centre*)) ) |
| S8 | S5 OR S6 OR S7 |
| S9 | S4 AND S8 |

Table 5. Grey Literature

| **Date** | **Source** | **URL** | **Exact Search String** | **Type of Material identified & number of each** |
| --- | --- | --- | --- | --- |
| August 25, 2023 | Google [2 pages reviewed] | Google.com | (policies OR policy OR procedure OR guideline OR BPG OR pathway) "social work" ("emergency department" OR "emergency room" OR "emergency ward") filetype:pdf | 4 – reports  2 - articles |
| August 25, 2023 | Google [2 pages reviewed] | Google.com | (policies OR policy OR procedure OR guideline OR BPG OR pathway) "social worker" ("emergency department" OR "emergency room" OR "emergency ward") filetype:pdf | 4 – reports |
| August 25, 2023 | Google [2 pages reviewed] | Google.com | (policies OR policy OR procedure OR guideline OR BPG OR pathway) "social services worker" ("emergency department" OR "emergency room" OR "emergency ward") filetype:pdf | 0 |
| August 25, 2023 | Google [2 pages reviewed] | Google.com | (policies OR policy OR procedure OR guideline OR BPG OR pathway) "social service worker" ("emergency department" OR "emergency room" OR "emergency ward") filetype:pdf | 0 |
| August 25, 2023 | Google [2 pages reviewed] | Google.com | (policies OR policy OR procedure OR guideline OR BPG OR pathway) "social work team" ("emergency department" OR "emergency room" OR "emergency ward") filetype:pdf | 0 |
| August 25, 2023 | Google [2 pages reviewed] | Google.com | (policies OR policy OR procedure OR guideline OR BPG OR pathway) "social care worker" ("emergency department" OR "emergency room" OR "emergency ward") filetype:pdf | 0 |
| August 25, 2023 | ProQuest Dissertations & Theses | <http://proxycheck.lib.umanitoba.ca/libraries/online/proxy.php?http://proquest.com?accountid=14569> | ("social worker" or "social services worker" or "social service worker" or "social work team" or "social care worker" or "social work") AND ((emergency or trauma) NEAR/2 (department* or unit* or ward* or hospital* or room* or center* or centre*) | 14 - theses |

| Appendix B. Data extraction of all included studies | | | | | | | | |
| --- | --- | --- | --- | --- | --- | --- | --- | --- |
| **Article Title** | **Authors** | **Year** | **Country** | **Study Type** | **Characteristics of social work role** | **Staffing** | **Themes** | **Consult/patient population** |
| Adapting the serious illness conversation guide for use in the emergency department by social workers | Aaronson, Emily Loving; Greenwald, Jeffrey L.; Krenzel, Lindsey R.; Rogers, Angelina M.; LaPointe, Lauren; Jacobsen, Juliet C. | 2021 | United States | Pilot study | - Social Workers used the adapted "Serious Illness Conversation Guide" to facilitate conversations about goals of care and clarify cardiopulmonary resuscitation (CPR) status in the emergency department | Not reported | Guiding patients through illness | Palliative and serious illness |
| Assessing the Acceptability and Feasibility of Leveraging Emergency Department Social Workers' Advanced Communication Skills to Assess Elderly Patients' Goals and Values | Aaronson, Emily Loving; Kennedy, Maura; Gillis-Crouch, Grace; Zheng, Hui; Jacobsen, Juliet; Ouchi, Kei et al. | 2023 | United States | Pilot study (Randomized clinical trial) | - Social Workers used the "Serious Illness Conversation" to facilitate a discussion on older adult patients' goals of care in the emergency department | Not reported | Guiding patients through illness | Older adults |
| The SCOPE intervention: Impact of a social care optimization pilot initiative in the emergency department | Adamson, Keith; Bliss, Rebecca; Shahab, Ramish; Sengsavang, Sonia; Craig, Shelly; Rankin, Vanessa, et al. | 2020 | Canada | Pilot study | - Social Workers worked to facilitate the Social Care Optimization Program in the Emergency (SCOPE) model in the emergency department   - The primary purpose of the Social Workers' role in SCOPE was "presceeening" patients' while they are being triaged and prior to being seen by a physician  - In this model the Social Workers would make recommendations to the ED physician about admission to hospital and also link patients' to appropriate community resources | Extended hours | Reducing hospital use and admissions  Connecting to community resources | Non-specific ED population |
| Social workers cut nonemergent visits 45% | Anonymous | 2007 | United States | Pilot study (Non-academic literature) | - Social Workers were placed in the ED with a specific mandate to connect patients under the "Horizon NJ Health" coverage plan with appropriate primary care  - In this intervention the Social Workers would scan the ED census for the "Horizon NJ Health" coverage patients, meet with them after they've been seen by a ED physician, discuss barriers to accessing primary care, and then connect them with the appropriate primary care for follow-up | Extended hours ("Peak hours") | Reducing hospital use and admissions  Connecting to community resources | Non-specific ED population |
| The value of the presence of social work in emergency departments | Auerbach, Charles; Mason, Susan E. | 2010 | United States | Quantitative study | - Social Workers supported ED patients at time of discharge and if possible assisted with a discharge plan which diverted patients from hospital admission | Not reported | Reducing hospital use and admissions  Disposition and safe discharge planning | Non-specific ED population |
| Implementing Interprofessional Alcohol Screening, Brief Intervention, and Referral to Treatment in the Emergency Department: An Evidence-Based Quality Improvement Initiative | Bacidore, Vicki; Letizia, MariJo; Mitchel, Ann M. | 2017 | United States | Pilot study | - Social Workers and nurses delivered the standardized alcohol screening, brief intervention, and referral to treatment (SBIRT) model in the ED  - Nurses completed the screening portion using the Alcohol Use Disorder Identification Test (AUDIT)and for any patients who screened positive the ED Social Worker was alerted and completed a brief intervention and referred patients to treatment | Not reported | Substance use and addiction support | Addictions |
| Effect of a Peer-Led Behavioral Intervention for Emergency Department Patients at High Risk of Fatal Opioid Overdose: A Randomized Clinical Trial | Beaudoin, Francesca L.; Jacka, Brendan P.; Li, Yu; Samuels, Elizabeth A.; Hallowell, Benjamin D.; Peachey, Alyssa M., et al. | 2022 | United States | Randomized clinical trial | - Social Workers were consulted to support patients who presented to the ED due to opioid use disorder (OUD)   - Social Workers (or peer recovery specialists in the trial) delivered a behavioural intervention within the ED that involved addressing the short-term and long-term needs of the patient, which could include supplying a take-home naloxone kit or connecting them with substance use treatment   - The Social Workers did not remain connected to the patients after discharge from the ED | Not reported | Substance use and addiction support | Addictions |
| Safe Discharge Needs Following Emergency Care for Intimate Partner Violence, Sexual Assault, and Sex Trafficking | Clery, Michael J.; Olsen, Eudora; Marcovitch, Hannah; Goodall, Harrison; Gentry, Jasmine; Wheatley, Matthew A., et al. | 2023 | United States | Quantitative study | - Social Workers were consulted to provide social support, as well as assisting with discharge plan to shelter for patients attending the ED due to forms of gender based violence (intimate partner violence, sexual assault and sex trafficking)  - Social Work was consulted after the patients received medical care  - If the Social Worker was unable to find a shelter space at time of discharge, patients were placed in the ED observation unit (EDOU) for further discharge planning support from Social Work | 24/7 | Disposition and safe discharge planning | Gender Based Violence |
| The Emergency Department Response to Women Experiencing Intimate Partner Violence: Insights From Interviews With Clinicians in Australia | Dawson, Angela J.; Rossiter, Chris; Doab, Anna; Romero, Bernadine; Fitzpatrick, Lesley; Fry, Margaret | 2019 | Australia | Qualitative study | - Social Workers were consulted by doctors and nurses in the ED, following medical treatment, when a women disclosed intimate partner violence  - Social Workers would address psychosocial and safety needs of patients and support clinical ED team to provide appropriate care  - Social Workers did not use specific screening tools but rather informal discussions with patients to address their needs  - Social Workers offered patients an opportunity to return to the ED if they continued to feel unsafe | Not reported | Psychosocial assessment and support  Supporting clinical team | Gender Based Violence |
| Social Work and the HIV Care Continuum: Assisting HIV Patients Diagnosed in an Emergency Department | Edmonds, Amy; Moore, Eric; Valdez, Andre; Tomlinson, Cheri | 2015 | United States | Quantitative study | - Social Workers were part of the Test, Educate, Support, and Treat Arizona (TESTAZ) HIV screening program in the ED  - Social Workers assisted the ED physician to deliver result notification to the patient  - Following result notification, the physician would leave and the Social Worker would stay with the patient and explain ongoing primary care, process of public health and partner notification, as well as offering handout on "what to expect"  - Social Worker would also refer patient to a patient navigator from a HIV primary care clinic | Not reported | Guiding patients through illness  Connecting to community resources  Supporting clinical team | HIV |
| Development and implementation of a model to improve identification of patients infected with HIV using diagnostic rapid testing in the emergency department | Haukoos, Jason S.; Hopkins, Emily; Eliopoulos, Vassily T.; Byyny, Richard L.; Laperriere, Kate A.; Mendoza, Myles X. et al. | 2007 | United States | Quantitative study | - Social Workers supported ED patients who were identified as being at-risk for undiagonosed HIV  - Social Workers provided HIV diagnostic pre-test and post-test counselling in the ED  - Pretest counselling included assessment of behavioural risks, overview of testing process, assessment of patients' psychological ability to receive results, assist in developing risk reduction plan and obtaining written consent for HIV diagnostic testing  - If test results were positive Social Work assisted the ED physician to deliver result notification, as well as providing crisis counselling and a referral to follow-up medical care  - Patients were also offered post-test counselling from Social Work if their results were negative | Not reported | Guiding patients through serious illness  Connecting to community resources  Supporting clinical team | HIV |
| Embedding an allied health service in the Nelson Hospital Emergency Department: A retrospective report of a six-month pilot project | Holmes, Clare; Hollebon, Debbie; Scranney, Alice; Exton, Hilary | 2016 | New Zealand | Pilot study | - Social Work was consulted either directly from triage or following assessment by ED physician, if the consult came directly from triage the Social Worker would have to liaise with the physician  - Social Work focused on patients who presented frequently with non-emergent concerns  - Social Work would identify patients' main issues and see how they could be better met by primary care, Social Work would also facilitate multidisciplinary team meetings and develop patient management plans | Weekdays | Reducing hospital use and admission  Care planning and case management | Frequent ED users |
| Feasibility of a screening tool for sex trafficking in an adult emergency department | Kaltiso, Sheri-Ann O.; Greenbaum, V. Jordan; Moran, Timothy P.; Osborne, Anwar D.; Korniotes, Jessica; Marazzi, Grace et al. | 2021 | United States | Quantitative study | - Patients were screened in ED for being at-risk for human trafficking  - If patients screened positive, Social Work was consulted and then interviewed the patient to determine their level of risk and whether they met the criteria for Trafficking Victims Protection Act (TVPA) definition for adult sex or labour human trafficking  - If patient met the criteria, the Social Worker assessed readiness for exit from human trafficking and assisted patient to access community resources, as well as to contact police | Not reported | Psychosocial assessment and support  Connecting to community resources | Gender Based Violence |
| A novel social work approach to emergency department buprenorphine induction and warm hand-off to community providers | Kelly, Timothy; Hoppe, Jason A.; Zuckerman, Matthew; Khoshnoud, Angela; Sholl, Benjamin; Heard, Kennon | 2020 | United States | Quantitative study | - Patients who were identified in the ED as having an opioid use disorder (OUD) were placed in a treatment pathway that automated a Social Work consult  - While patients were stabilized on buprenorphine in ED, Social Worker assessed potential treatment barriers and identified likelihood of success in outpatient treatment   - Social Work then determined most appropriate outpatient treatment and provided warm handoff to clinic | Extended hours | Substance use and addiction support  Connecting to community resources | Addictions |
| National Implementation of Emergency Department-Based Follow-up Program for Suicidal Attempts | Kim, Tae Han; Song, Kyoung Jun; Shin, Sang Do; Hong, Ki Jeong; Lee, Jung Kyu | 2022 | Korea | Quantitative study | - Social Workers positioned in the ED to scan for patients who have attempted suicide or are self harming  - While in the ED, Social Workers complete a screening interview and discuss community follow-up with patient  - Social Workers follow patients for a few weeks post-discharge from ED  - Social Workers will complete 4 sessions with patient either face-to-face or over the phone, during this time they will discuss medication compliance, wellbeing and outpatient scheduling  - Once the 4 sessions are complete, Social Workers will connect the patient with a community mental health centre for further support | Extended hours | Psychosocial assessment and support  Connecting to community resources  Care planning and case management | Mental Health |
| Domestic and family violence, non-lethal strangulation and social work intervention in the emergency department | Marks, Jacqueline; Markwell, Alexandra; Randell, Thomas; Hughes, James | 2020 | Australia | Quantitative study | - Patients who presented to the ED with injuries related to either domestic and family violence, as well as non-lethal strangulation were referred to Social Work  - Social Work was available 24/7 to review these cases and deliver intervention | 24/7 | Psychosocial assessment and support | Gender Based Violence |
| Factors related to admissions to a psychiatry unit from a medical emergency room: The role of social work | Mason, Susan E.; Auerbach, Charles | 2009 | United States | Quantitative study | - Social Workers in the ED collected patient data on demographics, insurance and function  - Social Workers met with patients' families to assess resources and willingness of family members to provide care at home  - Social Workers assisted in assessing patients for psychiatric symptoms and initiated referrals where appropriate  - Social Workers provided information from assessment to interdisciplinary ED team to determine patients' disposition at discharge | Not reported | Reducing hospital use and admission  Psychosocial assessment and support  Disposition and safe discharge planning | Mental Health |
| Emergency department social work in the UK and Sweden: evaluation by older frequent emergency department attenders | McLeod, E.; Olsson, M. | 2006 | Sweden and the United Kingdom | Qualitative study | Sweden:   - Social Workers received referrals from ED medical or nursing staff  - Social Workers primarily were responsible for bereavement counselling, provision of home care, cases complex social issues, and general care planning  UK:   - Social Workers were part of the multidisciplinary ED rapid response assessment team  - For all patients aged 65 or older, Social Workers contributed to a multidisciplinary assessment once the patient was medically stable  - Assessments were completed to avoid unnecessary hospital admission and to ensure patients were connected to appropriate services | Weekdays | Reducing hospital use and admission  Connecting to community resources | Older adults   Frequent ED users |
| From Their Perspective: The Connection between Life Stressors and Health Care Service Use Patterns of Homeless Frequent Users of the Emergency Department | Moore, Megan; Conrick, Kelsey M.; Reddy, Ashok; Allen, Ann; Jaffe, Craig | 2019 | United States | Qualitative study | - Social Workers work as part of the ED High User Case Management (HUCM) team  - As part of the HUCM team, Social Workers offer unhoused patients with four or more ED visits with in 6 months, short-term intensive case management | Not reported | Care planning and case management | Unhoused  Frequent ED users |
| When high pressure, system constraints, and a social justice mission collide: A socio-structural analysis of emergency department social work services | Moore, Megan; Cristofalo, Margaret; Dotolo, Danae; Torres, Nicole; Lahdya, Alexandra; Ho, Leyna | 2017 | United States | Qualitative study | - Social Work held a number of roles in the ED including, investigator, gatekeeper, resource broker, care coordinator, problem solver, crisis manager, advocate, discharge planner | 24/7 | Did not speak to specific social work role, discussed broad scope of social work practice in the ED | Non-specific ED population  *Authors initially intended to focus on patients with TBI, though Social Work participants indicated that a focus on this population did not accurately reflect their common practice |
| The Role of Social Work in Providing Mental Health Services and Care Coordination in an Urban Trauma Center Emergency Department | Moore, Megan; Whiteside, Lauren K.; Dotolo, Danae; Wang, Jin; Ho, Leyna; Conley, Bonnie | 2016 | United States | Quantitative study | - Social Workers received referrals from ED staff directly via pagers  - Social workers prioritized consults related to trauma and assault, homelessness, substance use and minors in the ED  - Interventions delivered by Social Work were largely mental health support, care coordination, material support, and crisis counselling | 24/7 | Did not speak to specific social work role, discussed broad scope of social work practice in the ED | Non-specific ED population |
| The emergency department social work intervention for mild traumatic brain injury (SWIFT-Acute): a pilot study | Moore, Megan; Winkelman, Amy; Kwong, Sharon; Segal, Steven P.; Manley, Geoffrey T.; Shumway, Martha | 2014 | United States | Pilot study | - After receiving medical treatment, patients who presented to the ED with a traumatic brain injury (TBI) were then referred to Social Work for further support through the Social Work Intervention for Mild Traumatic Brain Injury (SWIFT-Acute)  - As part of SWIFT-Acute, Social Workers completed a 10-20 minute intervention with patients that included education about TBI symptoms, decreasing alcohol use, coping strategies, reassurance, recovery process, as well as being informed of community and medical resources for follow-up | Not reported | Guiding patient through illness  Connecting to community resources | TBI |
| Patient care plans: an innovative approach to superusers in the emergency department | Pugh, L.; Duffy, L.; Stauss, M. | 2010 | United States | Pilot study (Case report in refereed journal) | - The Social Work and nurse in the ED created multidisciplinary care plans that addressed social, nursing and behavioural needs of patients who frequently presented to ED  - In addition, the ED Social Worker also assisted these patients in obtaining medical insurance, as well as other benefits, and connected them to appropriate community resources | Not reported | Connecting to community resources  Care planning and case management | Frequent ED users |
| Arranging Hospice Care from the Emergency Department: A Single Center Retrospective Study | Rege, Rahul M.; Peyton, Kelee; Pajka, Sarah E.; Grudzen, Corita R.; Conroy, Mark J.; Southerland, Lauren T. | 2022 | United States | Quantitative study | - ED Social Worker and case manager were involved in disposition planning for patients requiring hospice services  - Social Worker (along with case manager) would arrange for transportation, hospital bed, facility placement, home care services, respiratory equipment, as well as any other appropriate supports  - The needs of the patient were identified in the ED and the Social Worker would communicate with the appropriate hospice services | 24/7 | Connecting to community resources  Disposition and safe discharge planning | Palliative and serious illness |
| Emergency Departments as the Health Safety Nets of Society: A Descriptive and Multicenter Analysis of Social Worker Support in the Emergency Room | Selby, Sasha; Wang, Dongmei; Murray, Eoin; Lang, Eddy | 2018 | Canada | Quantitative study | - Social Workers in the ED were primarily consulted to assist with financial concerns, discharge planning, illness adjustment, addiction issues, and resource counseling | Not reported | Did not speak to specific social work role, discussed broad scope of social work practice in the ED | Non-specific ED population |
| Emergency Department staff experiences of screening and response for intimate partner violence in a multi-site feasibility study: Acceptability, enablers and barriers | Spangaro, Jo; Vajda, Jacqualine; Klineberg, Emily; Lin, Sen; Griffiths, Christine; McNamara, Lorna et al. | 2022 | Australia | Pilot study (Multi-method study) | - Women presenting to the ED after experiencing abuse were referred to Social Work for psychosocial support, risk assessment, safety planning and referrals   - This service was available 24/7 and was to be delivered within an hour of abuse disclosure  - During after-hours, Social Work was notified through an on-call roster | Extended hours | Psychosocial assessment and support | Gender based violence |
| The Role of Emergency Room Social Worker: An exploratory study | Elizabeth Fusenig | 2012 | United States | Qualitative study | - Social Workers primary services were psychosocial assessments, addictions support, counselling, reporting abuse, connecting to community resources, cultural brokering, as well as providing education to ED staff | Not reported | Did not speak to specific social work role, discussed broad scope of social work practice in the ED | Non-specific ED population |
